# Escherichia coli Nissle 1917 Protects Intestinal Barrier Function by Inhibiting NF-*κ*B-Mediated Activation of the MLCK-P-MLC Signaling Pathway

**DOI:** 10.1155/2019/5796491

**Published:** 2019-07-03

**Authors:** Shihao Guo, Shanwen Chen, Ju Ma, Yongchen Ma, Jing Zhu, Yuanyuan Ma, Yucun Liu, Pengyuan Wang, Yisheng Pan

**Affiliations:** Division of General Surgery, Peking University First Hospital, Peking University, Beijing 100034, China

## Abstract

Escherichia coli Nissle 1917 (EcN), a kind of probiotic, has been reported to have a protective effect on the intestinal barrier function and can ameliorate certain gastrointestinal disorders. In this study, the potential protective effect of EcN on the intestinal barrier function in a septic mouse model induced by cecal ligation and puncture (CLP) operation was investigated. FITC-Dextran 4,000 Da (FD-4) flux and the expression levels of tight junction (TJ) proteins were measured to evaluate the protective effect of EcN on the intestinal barrier function. Then, Caco-2 monolayers were utilized to further investigate the protective effect of the EcN supernatant (EcN^sup^) on the barrier dysfunction induced by TNF-*α* and IFN-*γ* in vitro; the plasma level of both the cytokines increased significantly during sepsis. Transepithelial electrical resistance (TEER) and FD-4 transmembrane flux were measured, and the localization of ZO-1 and Occludin was investigated by immunofluorescence. The expression of MLCK and the phosphorylation of MLC were detected by western blot. The activation of NF-*κ*B was explored by immunofluorescence, and CHIP assays were performed to investigate the conjunction of NF-*κ*B with the promoter of MLCK. The results indicated that EcN protected the intestinal barrier function in sepsis by ameliorating the altered expression and localization of TJ proteins and inhibiting the NF-*κ*B-mediated activation of the MLCK-P-MLC signaling pathway which might be one of the mechanisms underlying the effect of EcN.

## 1. Introduction

As a life-threatening disorder caused by acute systemic infection, sepsis remains as an important cause of death in critically ill patients [1]. Despite advanced development in the research of sepsis, the mortality rate among sepsis patients remains high. Because sepsis is a heterogeneous disease, people with sepsis always experience multiple organ dysfunction, including the lung, central nervous system, cardiovascular system, liver, kidney, and intestinal injury [2]. The gut, which consists of epithelial cells, immune systems, and microbiota, was assumed to be the promotor of this critical disease. Studies suggested that the intestinal barrier dysfunction propagated the development of multiple organ failure (MOF) in sepsis [3, 4].

The intestinal barrier system is the first line of defense which protects the gut from luminal pathogens and foodborne antigens. Broadly speaking, the normal intestinal barrier consists of the epithelial barrier, immune barrier, mucus barrier, and microecological barrier [5, 6]. In the structures regulating intestinal barrier properties, tight junctions localized in the apical contact area of adjacent enterocytes play a crucial part [7]. For example, ZO-1, a membrane-associated cytosolic protein, can form an anchor for different types of transmembrane proteins, such as Occludin and Claudins, to function as scaffold proteins in the constitution of TJ strands [8]. Claudin-1 is linked with sealing function and specific channel function, while Cluadin-2 controls the movement of ions and enhances transepithelial water flux [9]. Therefore, the tissue structure and physiological functions of TJs depend on the composition and interaction of these TJ proteins, as well as scaffold proteins, cytoskeleton related proteins, and regulatory components.

EcN, as a probiotic bacterium, has been applied for many years in the treatment of multiple gastrointestinal disorders including diarrhea and inflammatory bowel disease. Clinical studies have shown that EcN can prevent the recurrence of ulcerative colitis [10, 11]. Accumulating evidence have shown that the therapeutic role of EcN in gastrointestinal disorders was mainly mediated by its protective effect on the intestinal barrier function, especially on the function of the intestinal epithelial TJs [12–14]. Traditionally, these effects were mainly determined using probiotic suspension. In recent years, components released by commensal bacteria during cultivation, especially the outer membrane vesicles (OMVs) from EcN, are receiving more and more attention as a key role in the signaling process of intestinal mucosa [15, 16]. It has been reported that OMVs released by probiotic EcN enter intestinal epithelial cells through clathrin-mediated endocytosis, thereby triggering host immune and defense responses [17]. Studies have shown that EcN OMVs can induce the expression of antimicrobial peptides and regulate the cytokine/chemokine responses of intestinal epithelial cells and immune cells [18]. These vesicles can also promote the upregulation of TJ proteins ZO-1 and Claudin-14, as well as the downregulation of Claudin-2 in the intestinal epithelial cell line, thus enhancing the intestinal barrier function and reducing intestinal permeability [19]. The soluble secreted TcpC proteins from EcN can also upregulate ZO-1 and Claudin-14 but had no effect on the transcriptional regulation of Claudin-2 [19].

Though the beneficial effect of EcN in some gastrointestinal disorders has been proved, few researches go into the effect of EcN in acute intestine injuries, and the mechanisms underlying the protective effect of EcN remain unclear. Therefore, we used a CLP-induced septic mouse model and Caco-2 monolayers to investigate the effect of EcN on the intestinal barrier function and the expressions as well as the distributions of TJ proteins of intestinal epithelial cells during sepsis. More importantly, the mechanism underlying the protective effect of EcN on the intestinal barrier function was investigated in our study.

Excessive inflammatory cytokines, including TNF-*α*, have been reported to induce functional impairment of the intestinal epithelial barrier [20, 21]. Although the underlying mechanisms are not fully elucidated, the localization of TJ proteins produced by the activation of the MLCL-P-MLC signaling pathway is believed to play an important role in TNF-*α* induced intestinal barrier dysfunction [22, 23]. NF-*κ*B signaling was found to regulate intestinal barrier function via upregulating of MLCK [24–26]. Myosin light-chain kinase (MLCK) phosphorylates MLC, which is a mediator of actin dynamics, thus leading to contraction of actomyosin cytoskeleton and disruption of TJ in the intestine [27]. The present study reveals that EcN protected the intestinal barrier function by inhibiting the NF-*κ*B-mediated activation of the MLCK-P-MLC signaling pathway.

## 2. Materials and Methods

### 2.1. Animals

Male C57BL/6J mice (8 weeks old, 20-22 g) were obtained from the Lab Animal Institute of Peking University Health Center (Beijing, China). All mice were acclimated for at least 7 days in a standardized temperature (25-28°C), humidity (50-60%), and light environment (12 h light/12 h dark) with free access to standard food and tap water before initiation of the experimental procedure. The experimental protocol was approved by the University Ethics Committee for the use of experimental animals and conformed to the Guide for Care and Use of Laboratory Animals.

### 2.2. EcN Culture

The probiotic strain EcN (serotype O6:K5:H1) was provided by Biovector (Biovector NTCC Inc., China). Bacterial cells were grown at 37°C in Luria–Bertani Broth (LB) with constant rotation (150 rpm). Growth was monitored by measuring the optical density at 600 nm. After the EcN had been grown to the required concentration, the bacterial suspension was centrifuged and the supernatant was removed. Then, the pellet of EcN was dissolved with saline, and the bacteria solution was used in the subsequent animal feeding trial.

### 2.3. EcN Feeding Trial

The mice were randomly divided into two groups, the medicated group and the control group. The mice were fed with a standard food and water ad libitum supplemented either with or without EcN every day (1 × 10^9^ colony-forming unit (CFU) was given by oral gavage in the EcN group; controls received saline). The amount of consumed food and water was recorded. After 2 weeks, CLP operation or sham operation was performed as described below.

### 2.4. Sepsis Model Induced by CLP

The mice after feeding trial were randomly divided into the sham group (saline prefeeding+sham operation, *n* = 10), EcN group (EcN prefeeding+sham operation, *n* = 10), CLP group (saline prefeeding+CLP operation, *n* = 10), and EcN+CLP group (EcN prefeeding+CLP operation, *n* = 10). The mice were anesthetized with 10% chloral hydrate (30 mg/kg, intraperitoneally), and a midline incision was made in the abdomen. In the CLP group and EcN+CLP group, the cecum was isolated and ligated at a point approximately 1 cm from the cecal tip; the isolated cecum was then punctured twice with a 20-gauge needle and then gently squeezed to extrude a small amount of feces from the perforation sites. In the sham group and EcN group, the cecum was exposed but neither ligated nor punctured. The cecum was then placed back into the peritoneal cavity, and the incision was sutured and closed in two layers. The mice were resuscitated finally with 1 mL of saline by intraperitoneal injection [28].

### 2.5. *In Vivo* Intestinal Paracellular Permeability Assay

Intestinal permeability of mice was measured as previously described with little modifications [29]. At 24 h postoperation, mice were administered intragastrically with FD-4 (150 mg/mL, Sigma, USA) for 4 *μ*L/g body weight. 400 *μ*L blood samples were collected at 4 h after the administered of FD-4. A Synergy H_2_ microplate reader (BioTek Instruments, USA) with 492 nm excitation and 520 nm emission filters was used to measure the serum fluorescence level. Concentration of FD-4 was measured using standard curve produced by continuous dilution of FD-4.

### 2.6. Preparation of Bacteria-Free Supernatant of EcN Suspension (EcN Supernatant)

As described previously, EcN was grown in 4 mL Luria-Bertani (LB) broth (Solarbio, USA) overnight at 37°C [9]. The next day, 20 mL LB broth was inoculated from overnight culture to an optical density (OD600 nm) of 0.4. Bacteria were cultured to an OD600 of at least 1 and were used for the preparation of a further culture, starting with an OD600 of 0.3. When an OD600 above 1 was reached, LB broth was removed by centrifugation at 5,000 ×g and the pellet was washed with DMEM medium without phenol red (Gibco, USA). The bacterial pellet was resuspended to an OD600 of 2. From this, a preparative culture was inoculated to an OD600 of 0.3 and EcN was grown in DMEM to an OD600 of 1.5. This preparative culture was centrifuged at 8,000 ×g for 15 min at 4°C. The supernatant was filtrated using Sterivex-GV 0.22 mm (Merck Millipore, USA) and afterwards concentrated using an Amicon Ultra-15 Centrifuge Filter Device (Merck Millipore, USA) with a molecular weight cutoff (MWCO) of 100 kDa. The protein concentration of the concentrated supernatant was determined using the BCA method (Thermo Scientific, USA).

### 2.7. Cell Culture

The human colonic cells lines of Caco-2 were from the American Type Culture Collection (ATCC). Cells were cultured in DMEM High Glucose (Gibco, USA) supplemented with 10% (*v*/*v*) fetal bovine serum (FBS), 25 mM HEPES, 1% nonessential amino acids, penicillin (50 U/mL), and streptomycin (50 U/mL). Cultures were incubated at 37°C in a 5% CO_2_ atmosphere. In order to grow on the filters, the high-density cells (1 × 10^5^ cells) were seeded on Transwell filters with an aperture of 0.4 mm size (Corning Incorporated, USA). 10 ng/mL TNF-*α* and 10 ng/mL IFN-*γ* (PeproTech, USA) were added to the basolateral chambers, and different doses (100, 300, and 600 ng/mL) of EcN^sup^ were added to the upper chambers of Transwells for different times.

### 2.8. Transepithelial Electrical Resistance (TEER) Measurements

A 12-well transwell system was used in the TEER measurements. In short, Caco-2 cells were seeded in the upper chamber of the transwell plate and an epithelial volt-ohm meter ERS-2 (Merck Millipore, USA) was used to measure the changes of TEER [30, 31]. About 21 days after the cell fusion, the epithelial resistance of Caco-2 monolayers reached at least 500 *Ω* cm^2^; then, the cells were incubated with different reagents as indicated. The electrical resistance values were measured until we got three similar measurements in a row. The values were corrected for the background resistance of the blank hole and converted to *Ω* cm^2^.

### 2.9. Paracellular Marker FD-4 Flux Measurements

Paracellular permeability was evaluated as previously described [32, 33]. Caco-2 monolayers were treated according to the above methods. Then, the cells were washed with PBS and incubated with Hank's balanced salt solution containing 1 mg/mL FD-4 solution for 2 h. The FD-4 flux was evaluated by 100 *μ*L of culture medium from the lower chamber. A Synergy H_2_ microplate reader (BioTek Instruments, USA) was utilized to measure the fluorescent signal by using 492 nm excitation and 520 nm emission filters. The standard curve made by FD-4 continuous dilution was used to determine the concentration of FD-4.

### 2.10. Quantitative Real-Time Polymerase Chain Reaction (RT-qPCR) Analysis

The TRIzol one-step extraction method was used to extract the total cellular RNA (Trizol reagent; Invitrogen, USA), and the RNA was reverse transcribed into cDNA using kit (Thermo Fisher, USA). RT-qPCR was implemented using a SYBR PCR master kit (Thermo Fisher, USA). Primers were listed 5′-3′ as follows: GAPDH: F—GCACCGTCAAGGCTGAGAAC, R—ATGGTGGTGAAGACGCCAGT; ZO-1: F—AGCCTGCAAAGCCAGCTCA, R—AGTGGCCTGGATGGGTTCATAG; Claudin-1: F—GCATGAAGTGTATGAAGTGCTTGGA, R—CGATTCTATTGCCATACCATGCTG; Clauin-2: F—ATCCTCTGCTTTTCCTGCCC, R—GAGCCTCTAGTTGCAAGGGG; Occludin: F—CTTTGGCTACGGAGGTGGCTAT, R—CTTTGGCTGCTCTTGGGTCTG; and MLCK: F—AGGAAGGCAGCATTGAGGTTT, R—GCTTTCAGCAGGCAGAGGTAA. For each sample, RT-qPCR reactions were performed in triplicate. The comparative threshold cycle (CT) method (2^-△△CT^) with GAPDH as the internal reference gene was used to calculate the RNA relative expression as fold alteration. The relative mRNA expression in the treated samples was confirmed as a fold increase compared with the control group.

### 2.11. Western Blot Analysis

The total proteins of Caco-2 monolayers were extracted by the method previously described [34]. The protein concentration of Caco-2 monolayers was detected by the BCA method (Thermo Scientific, USA), and the extracts containing equal amounts of proteins (25 *μ*g) were electrophoresed in 6% or 10% polyacrylamide gel. The isolated proteins were then transferred onto a PVDF membrane. The membrane was blocked for 1 h at room temperature for nonspecific binding (5% bovine serum albumin (BSA) in TBS-Tween 20 buffer), then incubated with rabbit anti-ZO-1 monoclonal antibody (1 : 1000 dilution, Invitrogen, USA), rabbit anti-Claudin-1 monoclonal antibody (1 : 1000 dilution, CST, USA), rabbit anti-Claudin-2 monoclonal antibody (1 : 1000 dilution, CST, USA), rabbit anti-Occludin monoclonal antibody (1 : 1000 dilution, Invitrogen, USA), rabbit anti-MLCK monoclonal antibody (1 : 1000 dilution, Abcam, UK), rabbit anti-MLC antibody (1 : 1000 dilution, CST, USA), and rabbit anti-GAPDH monoclonal antibody (1 : 1000 dilution, CST, USA) at 4°C for the night. The membrane was then incubated with secondary antibodies at room temperature for 1 h, and blots were performed using ECL detection reagents (Merck Millipore, USA).

### 2.12. Immunofluorescence (IF) of ZO-1, Occludin, and NF-*κ*B p65 in Caco-2 Monolayers

Caco-2 monolayers were treated as indicated above; then, the localization of TJ protein ZO-1, Occludin, and NF-*κ*B p65 in cells was evaluated by immunofluorescence as described previously [26]. In short, filters were washed with PBS and fixed in 100% methanol at -20°C for the night, then fixed by 100% acetone at -20°C for 1 min. Next, filters were washed with PBS and blocked with 1% BSA at room temperature for 2 h. Then, the filters were incubated with anti-rabbit ZO-1 (1 : 100 dilution, Invitrogen, USA) and anti-mouse Occludin (1 : 200 dilution, Invitrogen, USA) or anti-rabbit NF-*κ*B p65 (1 : 100 dilution, CST, USA) antibody at 4°C for the night. After PBS washing, goat anti-rabbit IgG conjugated to Alexa488 (Molecular Probes, USA) and goat anti-mouse IgG conjugated to Alexa555 (Molecular Probes, USA) were used to incubate with filters in 1% BSA at room temperature for 1 h. Then, the cells were washed with PBS and subsequently stained with sodium iodide. After PBS washing, the Prolong Gold anti-fade reagent (Molecular Probes, USA) was used to mount cells; then, the cells were stored at 4°C in the dark until analyzed. The fluorescence was visualized under a Fluoview 1000 confocal microscope (Olympus, Japan).

### 2.13. ChIP Assay

Cells were treated with or without EcN^sup^ in the presence or absence of 10 ng/mL TNF-*α* and 10 ng/mL IFN-*γ* for 4 h before fixation. ChIP assays were performed as described previously with rabbit anti-p65 antibodies (1 : 1000 dilution, CST, USA) and rabbit anti-IgG antibodies served as the negative control. The assays were quantified by real-time PCR using primers flanking the *κ*B sites in the promoter of the MLCK gene. Primers were listed 5′-3′ as follows: *κ*B-1: F—GCTGCCTCTGCTGCAGTTCA, R—ACACACAGCTCCCCTCTCTG and *κ*B-2: F—CAAAGTGTCCCTCAAAGTGTC, R—TCACCCAGCCTCAGGTATTT.

### 2.14. Statistical Analysis

Two-tailed Student's *t*-test (unpaired) was used to analyze differences in mean values between groups (GraphPad Prism version 7.0 for Windows, GraphPad Software, USA). A *P* value < 0.05 was used to indicate the statistical significance. All results were expressed as the means ± standard error of the mean (SEM), and all experiments were repeated at least three times to ensure the reproducibility. Error bars were added to show standard deviation.

## 3. Results

### 3.1. EcN Protects the Intestinal Barrier Function in Septic Mice

**Figure 1:**
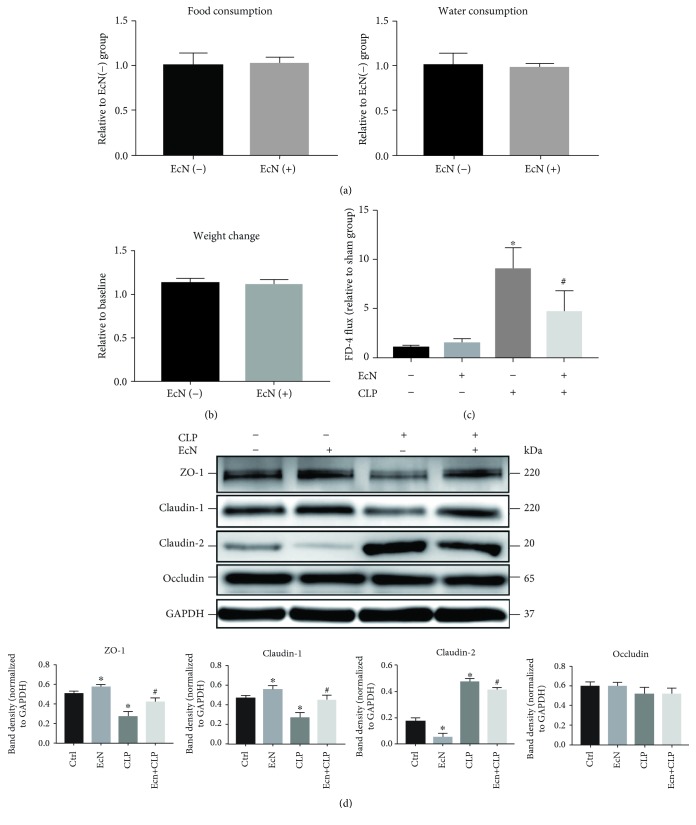
The effect of EcN on mice with sepsis induced by CLP operation. (a) Food and water consumptions during the 14 days of oral gavage with saline or EcN (1 × 10^9^ CFU/day). (b) The weight of mice after 14 days of oral gavage with saline or EcN (1 × 10^9^ CFU/day). (c) FD-4 flux at 24 h after sham operation or CLP operation. (d) The mucosal cells of a mouse intestine were collected at 24 h after sham operation or CLP operation, and the total proteins were harvested followed by western blotting assays. Results were expressed as the mean ± SEM (*n* = 10). ^∗^*P* < 0.05 vs. the sham group. ^#^*P* < 0.05 vs. the CLP group.

The mice were treated with PBS or EcN (1 × 10^9^ CFU/day) for 14 days. As shown in Figures 1(a) and 1(b), there were no significant differences on food consumption, water consumption, and body weight change between the two groups.

At the day 15, half of the mice from the two groups underwent CLP operation, and the other half of the mice underwent sham operation. After 24 h, the FD-4 fluxes of the intestine were tested (Figure 1(c)). There was no significant difference on the levels of serum FD-4 between the sham group and EcN group. The mice in the CLP group revealed an increase on serum FD-4 by about 8 times compared with those in the control group, suggesting that the intestinal barrier function of mice in CLP group was severely injured. Meanwhile, serum FD-4 of the mice in the EcN+CLP group decreased significantly compared with that of the mice in the CLP group. These data indicated that EcN could improve the function of intestinal epithelial barrier during sepsis.

### 3.2. EcN Ameliorates the Decreased Expression of TJ Proteins Induced by Sepsis in the Intestine of Mice

The expression levels of ZO-1, Claudin-1, Claudin-2, and Occludin in the small intestine of mice were tested by western blot (Figure 1(d)). Pretreatment with EcN increased the expression of ZO-1 and Claudin-1 and decreased the expression of Claudin-2 in mice's intestine compared to sham operation (*P* < 0.05), while the CLP operation significantly reduced the expression levels of ZO-1 and Claudin-1 and increased the expression of Claudin-2. Moreover, the ZO-1 and Claudin-1 expression in the intestine of mice that pretreated with EcN and then accepted CLP operation increased significantly, and the Claudin-2 expression decreased significantly compared with the CLP group. These results indicated that EcN could increase the expression of ZO-1 and Claudin-1 and decrease the expression of Claudin-2 in the intestine of mice. Meanwhile, the pretreatment of EcN can also ameliorate the change of TJ protein expression induced by sepsis.

### 3.3. EcN^sup^ Preserves the Intestinal Epithelial Barrier Function from TNF-*α*/IFN-*γ*-Induced Injury

**Figure 2:**
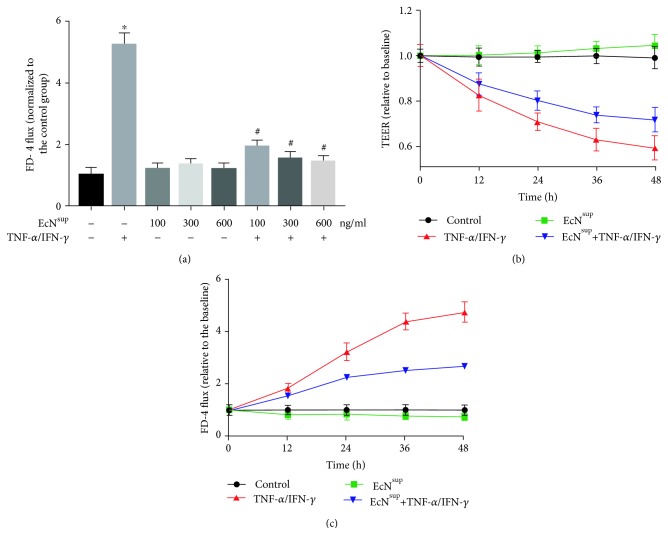
EcN^sup^ attenuated the injury of intestinal barrier function induced by TNF-*α*/IFN-*γ*. (a) Caco-2 monolayers were incubated with or without 10 ng/mL TNF-*α* and 10 ng/mL IFN-*γ* in the presence or absence of 100, 300, and 600 ng/mL EcN^sup^ for 48 h. EcN^sup^ significantly attenuated the increase of FD-4 flux induced by TNF-*α*/IFN-*γ* treatment. (b) Caco-2 monolayers were incubated with or without 10 ng/mL TNF-*α* and 10 ng/mL IFN-*γ* in the presence or absence of 100 ng/mL EcN^sup^ for 48 h. EcN^sup^ significantly attenuated TEER reduction induced by TNF-*α*/IFN-*γ* treatment since 12 h. (c) Caco-2 monolayers were treated as described in (b). EcN^sup^ significantly attenuated the increase of FD-4 flux induced by TNF-*α*/IFN-*γ* treatment since 12 h. Results were expressed as the mean ± SEM (*n* = 3). ^∗^*P* < 0.05 vs. control. ^#^*P* < 0.05 vs. TNF-*α*/IFN-*γ*.

Some reports have shown that the supernatant from the EcN culture could regulate the expression of cell tight junction proteins [9]. In order to investigate the protective effect of EcN supernatant (EcN^sup^), increasing doses of EcN^sup^ (100, 300, and 600 ng/mL) were added simultaneously with or without 10 ng/mL TNF-*α*/IFN-*γ* to Caco-2 monolayers for 48 h. As shown in Figure 2(a), there were no significant changes in FD-4 flux after the monolayers were treated with EcN^sup^ alone. However, EcN^sup^ ameliorated the elevated FD-4 flux induced by TNF-*α*/IFN-*γ* in a dose-dependent manner with EcN^sup^ at 600 ng/mL producing the strongest effect (*P* < 0.05). In the following experiments, we chose the dose of 100 ng/mL to verify the effect of EcN^sup^.

The permeability of monolayers was evaluated by both TEER and FD-4 flux (Figures 2(b) and 2(c)). As shown in Figure 2(b), after incubation with 10 ng/mL TNF-*α*/IFN-*γ* for 12 h, the TEER of Caco-2 monolayers decreased significantly (*P* < 0.05). After incubation for 48 h, the TEER of monolayers decreased to less than 60% relative to the baseline. As for the group with EcN^sup^ added simultaneously with 10 ng/mL TNF-*α* and 10 ng/mL IFN-*γ*, the decrease of TEER was ameliorated. At 24 h, the TEER was significantly higher compared with that of the TNF-*α*/IFN-*γ* group. After incubation for 48 h, the TEER became 70% relative to baseline, which was 17% higher than that of the TNF-*α*/IFN-*γ* group (*P* < 0.05).

The changes of FD-4 flux within 48 h were consistent with that of TEER (Figure 2(c)). After 12 h of treatment with 10 ng/mL TNF-*α* and 10 ng/mL IFN-*γ*, FD-4 flux increased significantly (*P* < 0.05) and rose to 5 times relative to baseline at 48 h. However, the group added simultaneously with EcN^sup^ as well as 10 ng/mL TNF-*α* and 10 ng/mL IFN-*γ* revealed a significant change since 24 h. After 48 h, the increase of FD-4 of Caco-2 monolayers with EcN^sup^ and TNF-*α*/IFN-*γ* was less than 3 times of the baseline, which was 40% lower than that of the TNF-*α*/IFN-*γ* group (*P* < 0.05).

### 3.4. EcN^sup^ Changes the Expression of ZO-1, Claudin-1, and Claudin-2

**Figure 3:**
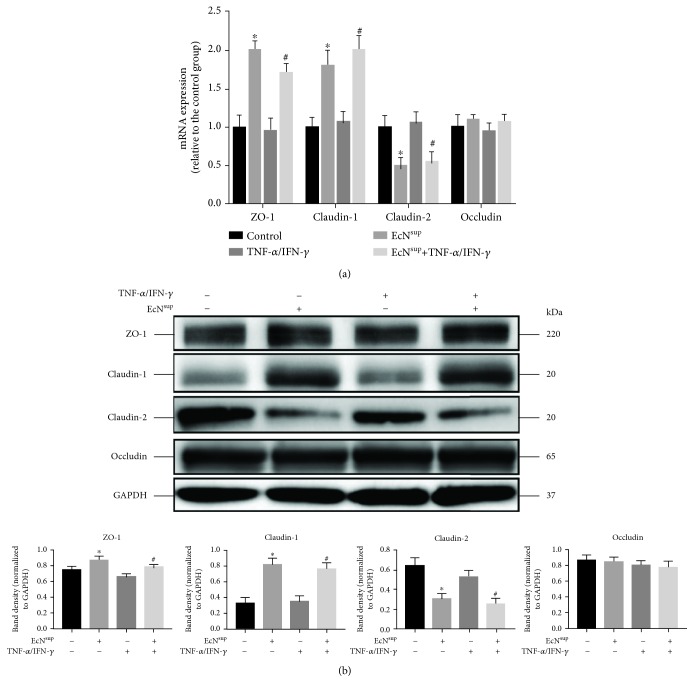
EcN^sup^ reversed the downregulation of TJ proteins induced by TNF-*α*/IFN-*γ* at both mRNA and protein levels. The total protein or RNA of monolayers was harvested after incubation for 48 h with or without 10 ng/mL TNF-*α* and 10 ng/mL IFN-*γ* in the presence or absence of 100 ng/mL EcN^sup^. The mRNA and protein level of ZO-1, Claudin-1, Claudin-2, and Occludin were tested by (a) RT-qPCR and (b) western blot, respectively. Results were expressed as the mean ± SEM (*n* = 3). ^∗^*P* < 0.05 vs. control. ^#^*P* < 0.05 vs. TNF-*α*/IFN-*γ*.

It has been reported that the injury of intestinal epithelial barrier function involves the changes of TJ protein expression level and the alteration of TJ protein localization [26, 35]. Therefore, we examined the effects of TNF-*α*/IFN-*γ* and EcN^sup^ on TJ protein expression levels on Caco-2 monolayers, which had been incubated for about 3 weeks after confluence and reached epithelial resistance of at least 500 *Ω* cm^2^, in the presence or absence of TNF-*α*/IFN-*γ* with or without EcN^sup^ treatment (Figures 3(a) and 3(b)). Compared with the control group, 10 ng/mL TNF-*α*/IFN-*γ* did not significantly change the expression levels of ZO-1, Claudin-1, Claudin-2, and Occludin within 48 h. However, simultaneous addition of EcN^sup^ in both groups showed increased expression levels of ZO-1 and Claudin-1 and decreased expression level of Claudin-2. As for the TJ protein Occludin, there were no significant changes among the treatments with or without TNF-*α* and IFN-*γ* in the absence or presence of EcN^sup^. The results showed that EcN^sup^ could increase the expression levels of ZO-1 and Claudin-1 and decrease the expression level of Claudin-2, thus reducing the permeability of monolayers. Considering the vital role of altered localization of TJs in the intestinal barrier injuries induced by TNF-*α*/IFN-*γ*, we set out to test the alterations of the distribution of TJs in Caco-2 monolayers from different groups.

### 3.5. EcN^sup^ Ameliorates TNF-*α*/IFN-*γ*-Induced Altered Localization of ZO-1 and Occludin by Inhibiting MLCK-P-MLC Signaling Pathway

**Figure 4:**
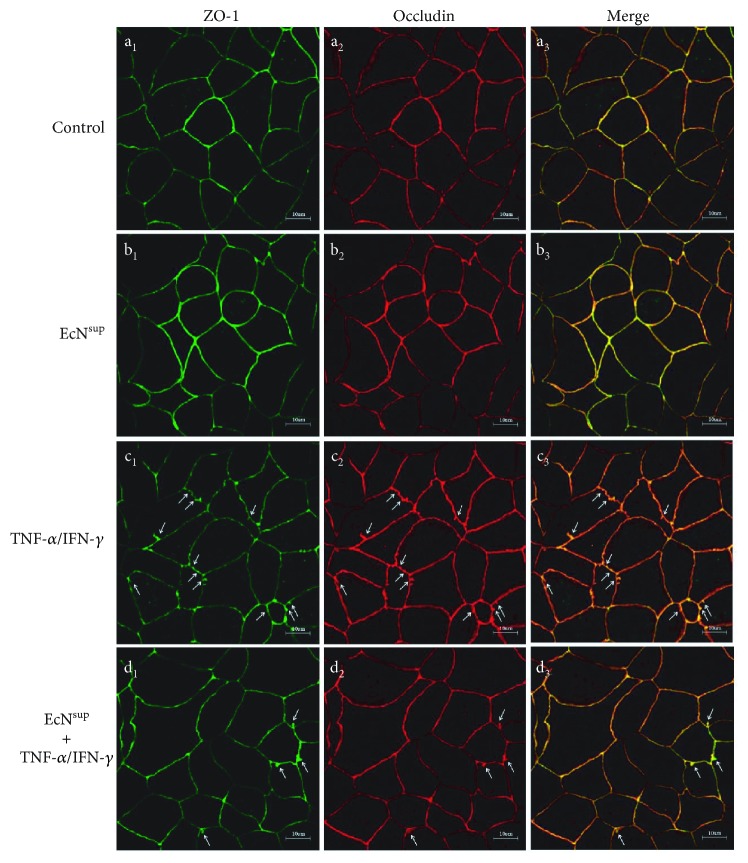
Immunofluorescence staining of Caco-2 cell monolayers grown on filters against ZO-1 and Occludin. (a_1_, a_2_, a_3_) In the control group, Caco-2 monolayers were treated with DMEM medium without TNF-*α*/IFN-*γ* or EcN^sup^. (b_1_, b_2_, b_3_) In the EcN^sup^ group, Caco-2 monolayers were incubated with EcN^sup^ for 48 h. (c_1_, c_2_, c_3_) In the TNF-*α*/IFN-*γ* group, Caco-2 monolayers were exposed to 10 ng/mL TNF-*α* and 10 ng/mL IFN-*γ* for 48 h. (d_1_, d_2_, d_3_) In the EcN^sup^+TNF-*α*/IFN-*γ* group, Caco-2 monolayers were incubated with 100 ng/mL EcN^sup^ for 48 h in the presence of 10 ng/mL TNF-*α* and 10 ng/mL IFN-*γ*. The medium containing different reagents was changed every 12 h.

In this study, ZO-1 and Occludin were selected as localization markers of TJ proteins (Figure 4). In normal Caco-2 monolayers, ZO-1 and Occludin immunofluorescence showed smooth edges and typical “chicken wire” shapes (Figure 4, a_1_, a_2_, and a_3_). Incubation with EcN^sup^ revealed the similar phenomenon (Figure 4, b_1_, b_2_, and b_3_). However, after being treated with 10 ng/mL TNF-*α*/IFN-*γ* for 48 h, the cell edges become serrated, the intercellular contact sites changed, and the ZO-1 network pattern was disrupted by the irregular labeling intensity; some parts are almost discontinuous. Occludin localization changes are similar to ZO-1 (Figure 4, c_1_, c_2_, and c_3_). These results suggested that TNF-*α*/IFN-*γ* resulted in the abnormal distribution of these TJ proteins. However, as shown, the addition of EcN^sup^ inhibited the localization changes of ZO-1 and Occludin induced by TNF-*α*/IFN-*γ*, and immunofluorescence remained a typical “chicken wire” pattern (Figure 4, d_1_, d_2_, and d_3_).

It has been reported that the MLCK-P-MLC pathway was closely related to the physiological and pathophysiological regulation of TJ protein localization [26, 36]. In consideration of the significant protective effect of EcN on the changes of TJ protein localization induced by TNF-*α* and IFN-*γ*, the influence of EcN on TNF-*α*/IFN-*γ* induced activation of the MLCK-P-MLC signaling pathway was further studied.

**Figure 5:**
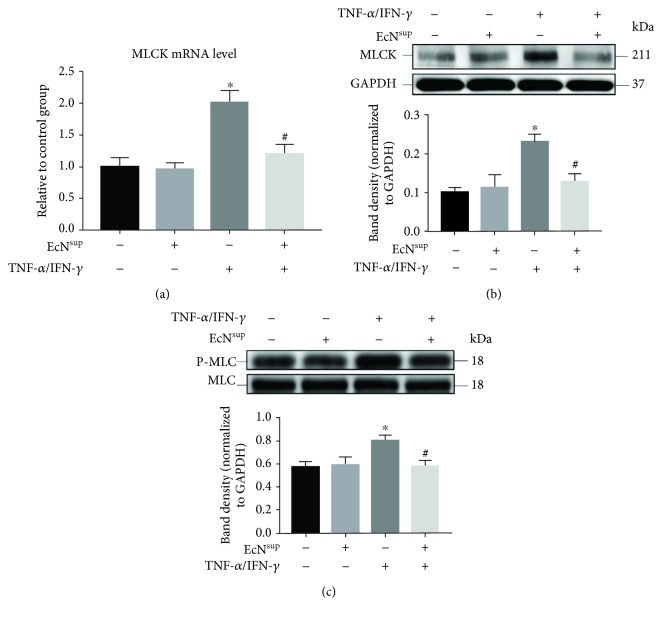
EcN^sup^ inhibited the increased expression of MLCK and the phosphorylation of MLC induced by TNF-*α*/IFN-*γ*. (a) The RNA of monolayers was harvested after incubation for 48 h in the absence or presence of 10 ng/mL TNF-*α* and 10 ng/mL IFN-*γ* with or without 100 ng/mL EcN^sup^, and the expression level of MLCK mRNA was detected by RT-qPCR. (b, c) The total protein of monolayers was harvested after treatment as described in (a), and the expression level of proteins was detected by western blot assays. Results were expressed as the mean ± SEM (*n* = 3). ^∗^*P* < 0.05 vs. control. ^#^*P* < 0.05 vs. TNF-*α*/IFN-*γ*.

The expression level of MLCK in Caco-2 monolayers was examined by RT-qPCR (Figure 5(a)) and western blot (Figure 5(b)), and the phosphorylation level of MLC was also examined by western blot (Figure 5(c)), after 48 h treatment with or without 10 ng/mL TNF-*α*/IFN-*γ* in the absence or presence of 100 ng/mL EcN^sup^. As shown in Figure 5, the expression levels of MLCK and phosphorylation levels of MLC were significantly increased after 48 h treatment with TNF-*α* and IFN-*γ* compared with the control group (*P* < 0.05). However, when cotreated with 100 ng/mL EcN^sup^, the expression of MLCK and the phosphorylation levels of MLC were significantly inhibited (*P* < 0.05). These results indicated that EcN^sup^ could alleviate the injuries of intestinal barrier function induced by TNF-*α* and IFN-*γ* by inhibiting the MLCK-P-MLC signaling pathway.

### 3.6. EcN^sup^ Inhibits the Activation of MLCK by Counteracting TNF-*α*/IFN-*γ*-Induced NF-*κ*B Activation

**Figure 6:**
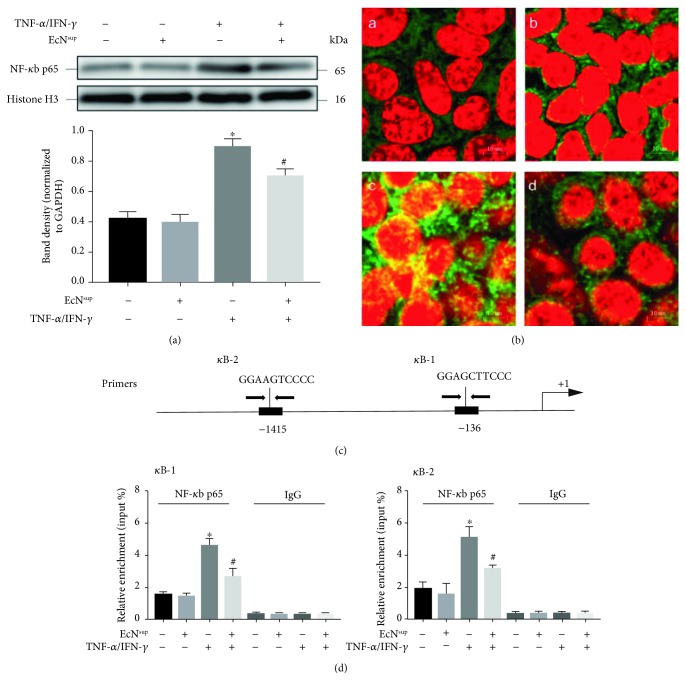
EcN^sup^ suppressed the activation of NF-*κ*B. (a) The nuclear protein of monolayers was harvested after incubation for 48 h in the absence or presence of 10 ng/mL TNF-*α* and 10 ng/mL IFN-*γ* with or without 100 ng/mL EcN^sup^ followed by western blot assays. (b) Caco-2 monolayers were stained for NF-*κ*B p65 by immunofluorescence. Cotreatment with 100 ng/mL EcN^sup^ significantly inhibited the nuclear translocation of NF-*κ*B p65 elicited by TNF-*α*/IFN-*γ*. A: The control group; B: Caco-2 monolayers were exposed to 100 ng/mL EcN^sup^ for 30 min; C: Caco-2 monolayers were incubated with 10 ng/mL TNF-*α* and 10 ng/mL IFN-*γ* for 30 min; and D: Caco-2 monolayers were incubated with 100 ng/mL EcN^sup^ for 30 min in the presence of 10 ng/mL TNF-*α* and 10 ng/mL IFN-*γ*. (c) Schematic illustration of human MLCK gene promoter containing two putative *κ*B sites (*κ*B-1 and *κ*B-2) at -136 and -1451 as indicated. The PCR primer locations were also indicated. (d) Caco-2 cells were pretreated for 4 h with or without 100 ng/mL EcN^sup^ in the absence or presence of 10 ng/mL TNF-*α* and 10 ng/mL IFN-*γ*. ChIP assays were performed with anti-p65 antibodies or IgG. The results for *κ*B-1 and *κ*B-2 sites are shown. Similar results were seen for 10 h treatment. Results were expressed as the mean ± SEM (*n* = 3). ^∗^*P* < 0.05 vs. control. ^#^*P* < 0.05 vs. TNF-*α*/IFN-*γ*.

Previous studies indicated that the increased expression of MLCK induced by TNF-*α*/IFN-*γ* was mediated by NF-*κ*B p65 signaling [22, 37]. According to the protective effect of EcN^sup^ mentioned above, the potential effect of EcN^sup^ on the activation of NF-*κ*B p65 elicited by TNF-*α*/IFN was further detected. As shown in Figure 6(a), the incubation with TNF-*α*/IFN-*γ* increased the nucleus translocation of NF-*κ*B p65. Although EcN^sup^ treatment alone did not affect the activation of NF-*κ*B p65, cotreatment with EcN^sup^ and TNF-*α*/IFN-*γ* reduced the increase of nucleus NF-*κ*B p65 induced by TNF-*α* and IFN-*γ*. Immunofluorescence of NF-*κ*B p65 showed that treatment with 10 ng/mL TNF-*α*/IFN-*γ* for 30 min could induce NF-*κ*B p65 aggregation into the nucleus in Caco-2 cells (Figure 6(b)). However, simultaneous addition of EcN^sup^ reduced the above phenomenon which was caused by TNF-*α* and IFN-*γ*.

In the further study, two putative *κ*B sites were identified in the MLCK promoter region (Figure 6(c)), which were predicted to mediate the stimulatory effect of TNF-*α* on MLCK expression. By ChIP assays, we confirmed that the two sites at -136 (*κ*B-1) and -1415 (*κ*B-2) were bound by NF-*κ*B p65 after TNF-*α*/IFN-*γ* treatment, but these TNF-*α*-induced p65 bindings were disrupted in the presence of EcN^sup^ (Figure 6(d)). These results suggested that the blocking of the activation of NF-*κ*B p65 signaling might be one of the mechanisms underlying the protective effect of EcN^sup^ on TNF-*α*/IFN-*γ* induced injury of intestinal epithelial barrier function.

## 4. Discussion

The intestinal barrier system is the first line of defense against the invasion of pathogens from the gut and a prerequisite for the stability of the intestine ecosystem. As the major connection between intestinal epithelial cells, the tight junctions on the surface of intestinal mucosa play an important role in maintaining mechanical integrity and normal functions of the intestinal mucosa [5]. In a variety of intestinal diseases, the injuries of tight junction played a crucial role in the initiation and progression of the diseases. Decreased expression and translocation of TJ proteins leads to the increase of intestinal permeability and thus facilitating the invasion of pathogens or toxic substances into the internal milieu [7].

In this study, we found that EcN could ameliorate the injuries of the intestinal barrier of CLP mice. These results showed that two weeks of EcN gavage significantly ameliorated the increase of intestinal FD-4 permeability of mice 24 h after CLP. In the subsequent western blot experiments, we found that CLP decreased the expression of TJ proteins ZO-1 and Claudin-1, while it increased the expression of Claudin-2 and MLCK in the intestinal epithelial cells of mice. The pretreatment of EcN not only improved the expression of ZO-1 and Claudin-1 and reduced the expression of Claudin-2 but also attenuated the alteration of expression of ZO-1, Claudin-1, Claudin-2, and MLCK caused by CLP. These results indicated that the colonization of EcN could improve the expression of intestinal mucosa of ZO-1 and Claudin-1, decrease the expression of Claudin-2, and protect the mechanical barrier of intestinal mucosa in the CLP septic mouse model.

In sepsis, increased plasma levels of inflammatory cytokines, including TNF-*α*, IFN-*γ*, IL-13, participated in damaging the intestinal barrier function [2]. In order to investigate the molecular mechanisms of the effect of EcN on the intestinal barrier function, Caco-2 monolayers were further utilized as the model of intestinal epithelia *in vitro*, and 10 ng/mL TNF-*α* and 10 ng/mL IFN-*γ* were added into the basolateral chambers of transwell plates to induce injuries of the monolayer's barrier function, mimicking the scenario in sepsis. Some reports have shown that the supernatant from the EcN culture could regulate the expression of cell tight junction proteins [9]. In order to verify the protective effect of EcN, the supernatant of EcN was further collected and used as the material for *in vitro* experiment.

Consistent with previous reports [22], after TNF-*α* and IFN-*γ* treatments, the TEER of Caco-2 monolayer decreased and FD-4 permeability increased, indicating that the barrier function of the Caco-2 monolayer was impaired. The addition of different doses of EcN^sup^ together with TNF-*α* and IFN-*γ* alleviated the changes of TEER and FD-4 significantly in 48 h. These findings indicated that EcN^sup^ had a protective effect on the injuries of monolayer barrier function caused by TNF-*α* and IFN-*γ*. It was reported that the expression and distribution of TJ protein were related to the changes of the barrier function of monolayers. Subsequently, the expression and distribution of TJ proteins were detected. The results of western blot and mRNA expression experiments showed that TNF-*α* and IFN-*γ* did not change the expression level of TJ protein, while EcN^sup^ enhanced the expression of ZO-1 and Claudin-11 and decreased the expression of Claudin-2. By immunofluorescence, we found that TNF-*α* and IFN-*γ* changed the distribution of TJ proteins on the surface of the cell membrane, while the cotreatment of EcN^sup^ ameliorated such damaging changes. These results suggested that EcN^sup^ protected the barrier function of the monolayer from the damage caused by TNF-*α* and IFN-*γ* through alleviating changes in the distribution of TJ proteins.

The MLCK-P-MLC signaling pathway has been widely reported to play an important role on the regulation of the distribution of TJ proteins both physiologically and pathologically. MLCK increase the phosphorylation of MLC, thus causing the changes in the distribution of TJ protein and the damage to the barrier function [25]. In the following experiments, we detected the activation of MLCK-P-MLC signals. The results suggested that TNF-*α* and IFN-*γ* treatment significantly increased the expression of MLCK and the phosphorylation of MLC. The cotreatment of EcN^sup^ inhibited the increase of MLCK expression caused by TNF-*α* and IFN-*γ*, as well as decreasing the phosphorylation of MLC. Actually, it has been found that the activation of MLCK-P-MLC signaling caused by TNF-*α* and IFN-*γ* was mediated by NF-*κ*B [38]. The protective effect of EcN^sup^ on the monolayer may be due to the inhibition of NF-*κ*B activation, so the activation of NF-*κ*B was detected in subsequent experiments. The results of western blot and the immunofluorescence experiment showed that EcN^sup^ inhibited the activation and nuclear translocation of NF-*κ*B p65 induced by TNF-*α* and IFN-*γ*. CHIP assays showed that TNF-*α* and IFN-*γ* increased the amount of NF-*κ*B binding to the promoter regions of MLCK, while the cotreatment of EcN^sup^ inhibited the increased binding caused by TNF-*α* and IFN-*γ*.

In summary, this study demonstrated for the first time that EcN protects the intestinal barrier function in sepsis both *in vivo* and *in vitro* and suggested that the inhibition of the NF-*κ*B-mediated MLCK-P-MLC signaling pathway might be one of the potential mechanisms of the protective effect of EcN.

## Data Availability

The data used to support the findings of this study are available from the corresponding author upon request.
